# Enlargement of the Prostate—III

**Published:** 1909-06-05

**Authors:** 


### ENLARGEMENT OF THE PROSTATE—III.

As a general rule it may be said that the best treatment for simple enlargement of the prostate is prostatectomy; at any rate if the condition has advanced to such a degree that there is residual urine in the bladder. In very early cases, however, much may be done towards relieving a patient's symptoms by enjoining on him a careful and well regulated existence. Thus his meals should be taken at a regular hour, his diet should be light, and anything likely to induce congestion of the prostate should be rigorously avoided; for this reason alcohol should only be taken in small quantities, and the total amount of fluid ingested should be restricted. The bowels should be regulated, and the bladder should never be allowed to become distended.

Such a course of life, though somewhat irksome in itself, will conduce to the patient's comfort, but it will not arrest the enlargement of the prostate, for this is a progressive condition, and sooner or later a pouch will form at the base of the bladder, and residual urine will collect. When this occurs the patient should have a catheter passed at regular intervals, to ensure that the bladder is completely empty from time to time. The frequency with which this should be done depends on the amount of residual urine present. Thus, if it is not more than four ounces it is sufficient to pass a catheter each night before retiring; but if it is as much as eight ounces a catheter ought to be passed after each act of micturition.

But it is obvious that this treatment is not ideal, apart from the inconvenience which it entails. Some patients, it is true, stand a catheter life fairly well, but others are very susceptible to instrumentation, and there is always the superadded risk that the patient if he passes a catheter for himself, may, sooner or later, damage the prostate and thus induce acute retention.

In the absence, therefore, of any gross contra-indication, an operation should always be advocated. The procedures on which reliance was placed before the present decade were most unsatisfactory. They consisted of such operations as castration, excision of a portion of the vas, or ligature of the internal iliac artery, the idea being that the prostate would atrophy. Such methods were empirical and unsatisfactory. But in the last ten years the operation of supra-pubic prostatectomy, which affords immediate and lasting relief, has replaced all other methods of operative treatment.

It is more important to consider the general condition of the patient before performing this operation tnan almost any other, on account of his advanced age. The state of the heart and the condition of the arteries should be examined, and the lungs auscultated for any sign of bronchitis. The urine should be carefully tested for abnormal constituents, and, as has already been mentioned, for deficiency of normal ones. Thus a twenty-four hours' specimen of the urine should be collected, and no operation should be performed if this contains less than 300 grains of urea. By observing these precautions the mortality of prostatectomy has been reduced from 25 per cent. to 10 per cent. in the last ten years.

The operation itself is not difficult. The bladder is first washed out with boracic or saline solution, until the fluid returns quite clear. Half a pint of fluid is left in the bladder. A median supra-pubic incision is then made. A dependent pouch of peritoneum is often found in front of the bladder. It contains omentum, and may easily be mistaken for the pad of fat which occupies the cavum Retzii; it should be pushed out of the way into the upper part of the wound. The bladder will then be seen. It will be easily recognised by the fasciculation of its walls owing to its muscular coat. A scalpel is plunged boldly into the viscus, and the contained fluid will escape. If the bladder has been adequately irrigated at the commencement of the operation, this will do no harm, as it should be quite aseptic. The opening in the bladder is enlarged in the axis of the trunk with a pair of scissors until it is large enough to admit three fingers. The interior of the bladder is next explored. In patients with a deep pelvis some difficulty may be experienced in getting down to the prostate satisfactorily. This can be overcome by the assistant putting his finger in the rectum and pressing the prostate forwards. A bougie should be passed into the bladder. The point of this will disclose the position of the internal opening of the urethra. The next step is to scratch through the mucous membrane of the bladder at the back of the prostate, where a vesical pouch has been formed. The finger nail is the best instrument for doing this. The prostatic capsule is thus exposed behind, and the organ can then be freed by passing the finger all round it. The last stage consists in freeing it anteriorly. This can only be done by dividing the urethra at the apex of the prostate. It has been said that the prostate can be enucleated without interfering with the continuity of the urethra; but the writer has never seen a case where this was possible except where one lobe of the prostate was occupied by an adenoma, and the whole organ was not removed. It is essential when freeing the organ to be sure that one is in the right layer, and experience alone will enable the operator to know this. Otherwise the veins in the prostatic plexus will be torn, and troublesome hæmorrhage may result.

In the actual enucleation the greatest difficulty is experienced when one is dealing either (i) with a prostate which is the subject of chronic inflammation, and is not very much enlarged, or (ii) with an enormously enlarged prostate. In the first case it is difficult to get into the proper layer on account of adhesions, and in the second the prostate may be so enlarged that its anterior surface is jammed against the back of the symphysis pubis, a condition which may render it very difficult to sweep the finger fully round it.

If there is any hæmorrhage after the removal of the organ it is best controlled by filling the bladder with hot saline solution. It is, of course, out of the question to attempt to ligature vessels in this situation. The wound in the bladder need not be sewn up. A large empyema tube is fixed in the supra-pubic wound with tapes, and the operation is complete.

The after treatment will be considered in a succeeding article.

